# Artificial visual environments to enhance mating success in colonised *Anopheles funestus*

**DOI:** 10.1186/s13071-026-07492-0

**Published:** 2026-06-08

**Authors:** Paul C. Mrosso, Ashley M. Burke, Halfan S. Ngowo, Megan A. Riddin, Fredros O. Okumu, Bernard W. T. Coetzee, Lizette L. Koekemoer

**Affiliations:** 1 Wits Research Institute for Malaria, Faculty of Health Sciences, University of the Witwatersrand, Johannesburg, South Africa; 2 Centre for Emerging Zoonotic and Parasitic Diseases, National Institute for Communicable Diseases of the National Health Laboratory Service, Johannesburg, South Africa; 3 Department of Environmental Health and Ecological Science, Ifakara Health Institute, Ifakara, Tanzania; 4 Faculty of Health Sciences, University of Pretoria Institute for Sustainable Malaria Control, University of Pretoria, Pretoria, South Africa; 5 School of Biodiversity, One Health and Veterinary Medicine (SBOHVM), University of Glasgow, Glasgow, UK; 6 School of Life Science and Bioengineering, The Nelson Mandela African Institution of Science and Technology, Arusha, Tanzania; 7 Conservation Ecology Research Unit, Department of Zoology and Entomology, University of Pretoria, Pretoria, South Africa

**Keywords:** *Anopheles funestus* rearing, Black horizons, Insemination rates, Rearing cage, Visual markers

## Abstract

**Background:**

Establishing and maintaining laboratory colonies of the malaria vector, *Anopheles funestus* using newly collected material has proven challenging, in part because of their low propensity to mate in captivity. In this study we assessed how cage conditions influence the mating success of two *An. funestus* strains originating from different geographic areas, Angola (FANG) and Mozambique (FUMOZ).

**Methods:**

The visual environment in adult mosquito-rearing cages was manipulated either by covering the cages in different planes with black opaque cloth (referred to as black horizons) or by placing black visual markers at various positions inside the cages. Mating success was assessed by dissecting the spermathecae capsule of the females after the standard 10-day mating period.

**Results:**

Insemination rates were consistently higher in the *An. funestus* FANG strain than in the FUMOZ strain in both the black horizon (odds ratio [OR] 0.31, 95% confidence interval [CI] 0.21–0.44, *p* < 0.001) and visual marker experiments (OR 0.12, 95% CI 0.08–0.19, *p* < 0.001). The inclusion of black horizons and visual markers significantly increased insemination in both strains (*p* < 0.001). However, strain-specific responses were evident: FANG showed significantly greater insemination rates in the side-covered cages (OR 2.06, 95% CI 1.41–3.01, *p* < 0.001), whereas FUMOZ insemination rates declined under the same condition (OR 0.41, 95% CI 0.24–0.70, *p* < 0.001). The insemination rate of the FUMOZ strain was significantly higher in top-covered cages (OR 0.59, 95% CI 0.38–0.95, *p* = 0.03) and when a visual marker was placed at the bottom of cage (OR 2.18, 95% CI 1.25–3.81, *p* = 0.006), while FANG insemination rates were unaffected by marker position.

**Conclusions:**

This study demonstrates that manipulating the visual environment within adult mosquito-rearing cages can significantly enhance mating success in *An. funestus*, although the effectiveness of specific visual cues varies between strains. While both FANG and FUMOZ responded positively to visual enhancements, their differing responses to the same conditions underscore the importance of tailoring rearing protocols as mating stimuli at their original location may be associated with specific environmental features. These findings offer preliminary guidance for improving the colonisation and maintenance of *An. funestus* in laboratory settings, while highlighting the need for further research to improve mating success for this species.

**Graphical Abstract:**

**Figure:**
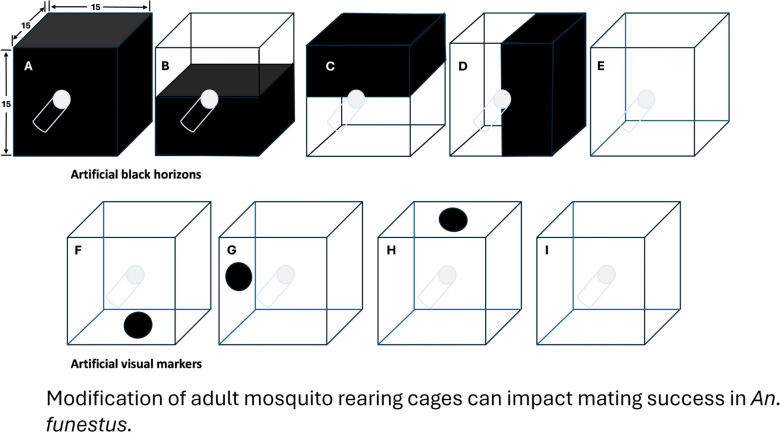

**Supplementary Information:**

The online version contains supplementary material available at 10.1186/s13071-026-07492-0.

## Background

*Anopheles funestus sensu stricto* (hereafter referred to as *An. funestus*) plays a major role in malaria transmission in Africa [1, 2]. There have been several attempts to establish sustained laboratory colonies of this species to support studies of its biology and control, but success has been limited [3, 4]. Previously, only two *An. funestus* strains, one from Angola (FANG) and the second from Mozambique (FUMOZ), had been successfully colonised in the laboratory from wild-caught populations [4]. However, more recently, a strain from Tanzania was also colonised [3]. The FANG and FUMOZ strains have been widely used in studies to understand (among other studies) the physiology and life history of *An. funestus* to enhance and improve rearing success under insectary conditions [3, 5, 6]. However, the low mating success in captivity has been identified as the main hindrance towards establishing and sustaining *An. funestus* in the laboratory [3].

Under natural conditions, *An. funestus* females, like other female *Anopheles* mosquitoes, mate with males in swarms [7]. Swarming activities are known to occur during sunset and at specific locations [7–9]. Various natural and artificial environmental features, such as vegetation, houses, hills, or open savanna fields, may create distinct light horizons (horizontal visual outlines), and these may stimulate swarming. In addition, surface or ground markers, such as soil colour and the presence or absence of objects, have been associated with *An. funestus* swarms [7–9]. Artificial lighting systems that mimic the natural photoperiod of 12:12 h (light:dark cycle), including a dusk and dawn period, have been used in *Anopheles* insectaries [10], but these conditions have not significantly improved the colonisation of species belonging to the *An. funestus* group compared to those in the *An. gambiae* complex [11]. Swarm induction has also been achieved both with simulated artificial horizons in laboratory cages in *An. gambiae* and *An. arabiensis* [12, 13], and contrasting visual markers, dimming lights, and other natural swarm markers in large semi-field experiment cages/chambers [13]. Covering cages with a black cloth reduces light intensity inside rearing cages and improves longevity and the mating of reared *An. arabiensis* [14]. However, no mosquito-rearing cage has yet been established that consistently ensures the successful mating of *An. funestus.*

Establishing conditions that will improve the mating success of *An. funestus* in rearing cages would be a major tool for enhancing its colonisation. In this study, we report on the impact of manipulating the environment inside adult rearing cages by adding an artificial horizon to mimic variation in the light horizon and contrasting visual markers on the mating success of two *An. funestus* strains that originated from different geographical locations.

## Methods

### Experimental site

Experiments were conducted in the Botha de Meillon insectary (BDMI) located at the National Institute for Communicable Diseases (NICD), Johannesburg, South Africa. The average insectary temperature was 26 ± 2 °C, with an average humidity of 70 ± 10%, and a 12:12 h light: dark (day: night) phase cycle. A set of three 42-W halogen bulbs (Pick n Pay, Cape Town, South Africa) was located on the ceiling; these gradually brightened over a 30-min period to simulate the onset of sunrise and dimmed over a 3-min period to simulate the onset of sunset, remaining lit during the day/light phase. Eight light-emitting diode (LED) tube lights (F58W/GRO-T8, Germany) were located beneath the rearing shelves to directly illuminate the larval rearing bowls (but not the adult cages) to enable researchers and laboratory staff to continue working (feeding larvae and harvesting adults) in the larval stages in the insectary. These lights switched on 30 min after sunrise and switched off 30 min before sunset.

### *Anopheles funestus* strains

The two strains of *An. funestus* used in this study were established in the early 2000s from field-collected mosquitoes from two geographical locations: Mozambique (established in 2000) (denoted as FUMOZ) and Angola (established in 2002) (denoted as FANG) [4, 15]. Although both the FANG and FUMOZ strains belong to the MW molecular lineage of *An. funestus*, they differ at the finer population structure level [11]. FANG belongs to clade I, while FUMOZ includes both clade I and clade II genotypes [11, 16]. For the present study, adult FUMOZ strains were obtained from a colony that was routinely reared in cubical BugDorm cages (30 cm) (MegaView Science Co. Ltd., Taichung, Taiwan). Adult FANG strains were obtained from a colony that was routinely reared in non-transparent white cylindrical polypropylene bucket cages (26.5 × 36.5 × 30.5 cm [height × base diameter × lid diameter]) with a clear fine mesh top cover and a round side hole covered by a translucent fine-meshed sleeve for transferring material into and out of the cage. Both FUMOZ and FANG adult mosquitoes were reared at a density of approximately 1,500–2,000 mosquitoes per cage and were provided a 10% sugar solution, which was changed twice weekly. Adult mosquitoes were blood-fed twice a week on anaesthetised guinea pigs, then egg plates were provided after at least two blood meals. Mosquito eggs were washed into a rectangular plastic larval rearing tub (12 × 20 × 7 cm) containing 750 ml of distilled water, where first- and second-instar larvae were reared. The third- to fourth-instar larvae were transferred at a density of about 300 larvae into white-coloured non-transparent plastic basins (15 × 35 × 13 cm) covered with a clear fine net. Larvae were maintained on a standard powdered food consisting of crushed dog biscuits (Beeno®; Martin & Martin (Pty) Ltd, Cape Town, South Africa; http://www.beeno.co.za) and brewer’s yeast (Vital®; Vital Health Foods, Cape Town, South Africa), mixed at a 3:1 ratio, respectively.

### Visual cues and cage alterations

**Fig. 1:**
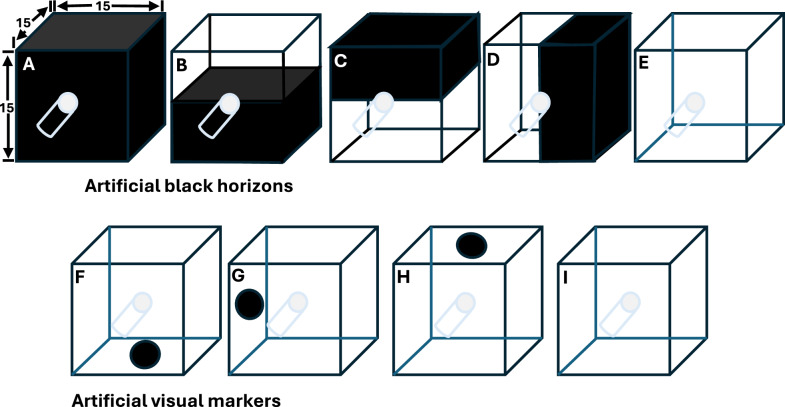
**A-E** Illustration of adult mosquito rearing cage with standard sleeve opening and black cloth coverings: **A** fully covered cage, **B** cage with bottom half covered on the horizontal plane, **C** cage with top half covered on the horizontal plane, **D** cage half covered on the vertical plane, **E** cage not covered (control cage).** F-I **Illustration of adult mosquito rearing cages with artificial visual markers: **F** swarm marker fixed onto the base of the cage, **G** swarm marker fixed onto one side of the cage, **H** swarm marker fixed onto the top of the cage, **I** no swarm marker (control cage)

Five experimental cages (15-cm cubes; MegaView Science Co. Ltd.) were covered with a black opaque cloth to create different covering planes: one cage was fully covered; two cages were covered on a horizontal plane covering the top-half or bottom-half (referred as top and bottom horizon, respectively); one cage was half covered on a vertical plane (referred as vertical horizon); and one cage completely uncovered to serve as a control (Fig. 1). Similarly, the effect of artificial visual markers were tested in a total of four experimental cages. In three cages, the black visual markers consisted of a round black paper card (7 cm in diameter) fixed either to the centre of the cage floor on the left hand side or to the top side; the fourth cage did not contain a black visual marker and served as the control (Fig. 1). Smaller experimental cages were used in order to reduce the number of adults required per cage (*n* = 100) compared to 1,500–2,000 individuals in larger cages; this design was not to mimic natural or true horizons and was purely used to manipulate visual contrast inside the experimental cages. Light intensity and spectral composition were not recorded inside the cage or in the room. However, all experiments were conducted in the same insectary room and under the same lighting conditions, ensuring that treatments were exposed to similar, consistent ambient illumination.

### Experiment setting

Male and female pupae were sorted under an Olympus dissecting microscope (SZX7, model SZ2-ILST; Olympus Corp., Tokyo, Japan). A total of 100 pupae of *An. funestus* FANG or FUMOZ strains, respectively, at a 1:1 male:female ratio, were sorted and placed in separate plastic bowls (diameter 12 cm, height 5 cm). The bowls containing 100 pupae (1:1 male: female ratio) were placed in separate experimental cages with access to a 10% sugar solution and left to emerge. Emerged adults were allowed to mate for 10 days in the same cage [6], and the sugar water provided was changed every 2 days. Six biological replicates of the experiment were conducted using different mosquito generation cohorts, and only one cage per biological replicate was set for each cage modification. To reduce the positional effect of cages, all experimental cages were randomly placed (the order of the cages differed between biological replicates) on the top shelf and illuminated at about an 80˚ angle from the dimming ceiling lights. On day 10, surviving females were removed from the cages and their spermatheca were dissected.

### Spermatheca dissection

Spermatheca dissections were performed as described in* Methods in Anopheles Research* [17], with the modification that mosquitoes were first knocked down in a paper cup covered with a net in a − 20 °C freezer for 10 min. Mosquitoes were mounted on a clean microscope slide with a drop of distilled water, and the last abdominal segment was removed using a pair of fine forceps under the stereo microscope to isolate the spermatheca. The remaining bodies were discarded, and spermathecae were observed under an Olympus light microscope (BX50, model BX50F4; Olympus Corp.) equipped with a 40× objective, with 400× magnification on the Stream Essentials 1.9.4 imaging software suite (Olympus Corp.). Spermatheca from females containing sperm were recorded as “mated”, while a clear spermatheca was scored as “not mated”.

### Data analysis

Insemination rates of *An. funestus* FANG and FUMOZ strains were analysed separately for the black horizon and visual marker experiments using generalised linear mixed models (GLMMs) with a binomial error distribution and logit link in R (version 4.3.3) with the *lme4* package (version 1.1–37) (R Foundation for Statistical Computing, Vienna, Austria). Mating success (inseminated vs. not inseminated) was modelled as a function of Cage modification (black horizon or visual marker position) and Strain, including their interaction, with experimental replicate fitted as a random intercept: Insemination ~ Cage specification × Strain + (1|Experimental replicate). The Significance of fixed effects (Cage modification, Strain, and their interaction) was assessed using likelihood ratio tests (LRTs) comparing full and reduced models (analysis of variance (ANOVA] test = “Chisq”). Pairwise comparisons of estimated marginal means were performed using the *emmeans* package with Tukey adjustments for multiple testing, and results are presented as odds ratios (ORs) with 95% confidence intervals (CIs) and corresponding *p*-values.

## Results

### The effect of black horizons on the mating success of* An. funestus*

**Fig. 2:**
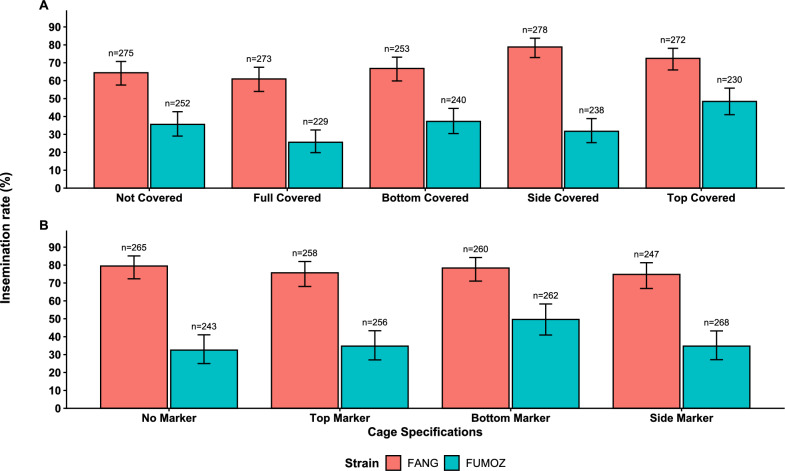
The insemination rates of* Anopheles funestus* FANG and FUMOZ strains under different adult mosquito holding cage modifications.** A** Artificial black horizon treatments,** B** artificial visual marker treatments

Insemination rates of the *An. funestus* strains FANG and FUMOZ were significantly affected by cage covering that created a black horizon (*χ*^2^_4_ = 34.5, *p* < 0.001), strain (*χ*^2^_1_ = 42.1, *p* < 0.001), and their interaction (*χ*^2^_4_ = 12.8, *p* = 0.012). The FANG strain exhibited higher insemination rates than FUMOZ across all cage conditions (OR 0.31, 95% CI 0.21–0.44, *p* < 0.001) (Table 1). Top-covered cages were associated with insemination in both strains, with no significant difference between them (OR 1.17, 95% CI 0.70–1.96, *p* = 0.554). Regarding the FANG strain specifically, side-covered cages produced the highest insemination rates (79%) when the interaction between the black horizon and strain was considered (OR 2.06, 95% CI 1.41–3.01, *p* < 0.001). In contrast, insemination rates in the FUMOZ strain remained lower overall (26–48%) but showed a significant increase in the top-covered cage compared with the uncovered cage (set as standard) when the interaction term was excluded (OR 0.59, 95% CI 0.38–0.95, *p* = 0.03; Additional file 1: Table S1; Fig. 2a).

**Table 1:** Odds ratios from the generalised linear mixed model showing the effects of black horizons, strain and their interaction on the insemination rates of *Anopheles funestus* FANG and FUMOZ strains

Predictor	Comparison	Odds ratio	95% Confidence interval	*p*-value^a^
FANG + not covered	Intercept	1.81	1.36–2.41	< 0.001***
FANG + full covered	FANG + not covered	0.86	0.61–1.22	0.406
FANG + bottom covered	FANG + not covered	1.11	0.78–1.60	0.560
FANG + side covered	FANG + not covered	2.06	1.41–3.01	< 0.001***
FANG + top covered	FANG + not covered	1.45	1.01–2.09	0.044*
FUMOZ + not covered	FANG + not covered	0.31	0.21–0.44	< 0.001***
FUMOZ + fully covered	FANG + fully covered	0.72	0.43–1.22	0.228
FUMOZ + bottom covered	FANG + bottom covered	0.97	0.58–1.62	0.893
FUMOZ + side covered	FANG + side covered	0.41	0.24–0.70	0.001**
FUMOZ + top covered	FANG + top covered	1.17	0.70–1.96	0.554

Reference categories: FANG (strain) + not covered (black horizon treatment). For the FANG strain, treatment effects are expressed relative to FANG + not covered. For the FUMOZ strain, odds ratios are expressed relative to the corresponding FANG treatment category shown in the Comparison column

^a^*p*-values denoted by asterisks indicate that Predictor had a significant effect on *Anopheles funestus* insemination rates at *p = 0.05, **p = 0.001 and ***p < 0.001

### The impact of a black visual marker on the mating success of* An. funestus*

Insemination rates of the *An. funestus:* FANG and FUMOZ strains were significantly influenced by strain type (*χ*^2^_6_ = 21.7, *p* < 0.001) and the interaction of strain type with the visual marker position (*χ*^2^_6_ = 334, *p* < 0.001), with a marginal interaction between visual marker position and strain (*χ*^2^_3_ = 7.7, *p* = 0.05). The FANG strain consistently maintained high insemination rates across all visual marker placements (Table 2; Fig. 2b), with no significant within-strain differences (Addition file 1: Table S2). In contrast, the FUMOZ strain exhibited lower insemination rates overall compared with the FANG strain (OR 0.12, 95% CI 0.08–0.19, *p* < 0.001), but achieved significantly higher insemination rates when the visual marker was positioned at the bottom of the cage (OR 2.18, 95% CI 1.25–3.81, *p* = 0.006 (Table 2; Fig. 2b).

**Table 2:** Odds ratios from the generalised linear mixed model showing the effects of the inclusion of visual markers, strain, and their interaction on the insemination rates of *Anopheles funestus* FANG and FUMOZ strains

Predictors	Comparison	Odds ratios	95% Confidence interval	*p*-value
FANG + no marker	Intercept	3.87	2.62–5.71	< 0.001***
FANG + top marker	FANG + no marker	0.81	0.53–1.22	0.307
FANG + bottom marker	FANG + no marker	0.94	0.61–1.43	0.759
FANG + side marker	FANG no marker	0.77	0.51–1.16	0.214
FUMOZ + no marker	FANG + no marker	0.12	0.08–0.19	< 0.001***
FUMOZ + top marker	FANG + top marker	1.37	0.78–2.40	0.267
FUMOZ + bottom marker	FANG + bottom marker	2.18	1.25–3.81	0.006**
FUMOZ + side marker	FANG + side marker	1.44	0.82–2.51	0.200

Reference categories: FANG (strain) + no marker (marker treatment). For the FANG strain, odds ratios for the experimental conditions top marker, bottom marker and side marker are expressed relative to the FANG + no marker experimental condition. For the FUMOZ strain, odds ratios are expressed relative to the corresponding FANG marker category shown in the Comparison column

^a^*p*-values denoted by asterisks indicate that Predictor had a significant effect on *Anopheles funestus* mating success at **p = 0.01 and ***p < 0.001

## Discussion

Attempts to improve conditions for the colonisation and maintenance of *An. funestus* colonies in captivity have been carried out in different locations and under varying experimental conditions [3, 4]. Maximising the mating success rates of *An. funestus* will complement existing larva rearing [5, 6] and adult feeding procedures to improve the physiological fitness of this vector in captivity [5, 6]. The findings from this study provide insight into the important modifications that can improve the mating rates of *An. funestus* in insectary rearing cages*.* Our results also show that the two strains of *An. funestus* used in this study mated at different rates under similar cage modifications.

The top covering of the cage to create a bottom horizon favoured mating of the FUMOZ strain, while the side covering favoured the FANG strain’s mating success rate. Considering the natural preference of *An. funestus* to rest in dark places in their habitats [18], the top and side cage coverings may have created a dark resting area relative to the ceiling lights of the insectary room, allowing mosquitoes to rest and reserve additional energy required for increased mating activities. Covering mosquito cages has been shown to improve mating and survival time in *Anopheles arabiensis* [14], possibly due to the dark space created by the cover being conducive to resting, consequently resulting in increased energy reserves [19]. Several studies have reported the importance of light phase changes for mosquito circadian clock-dependent behaviour, including mating [20]. The limited light-to-dark contrast inside the fully covered cage (including reduced light-to-dark contrast that promotes swarming and mating) may have resulted in the lowest mating success recorded for both strains compared with other cage modifications evaluated in the present study. This result demonstrates the importance of light in the environment for the mating success of *An. funestus*.

Likewise, the addition of black visual markers in adult *An. funestus* cage significantly improved the mating success of the FUMOZ strain, while not having any notable impact on the mating of the FANG strain compared to the controls. The black visual marker placed on the base of the cage might have induced swarming activity by providing contrast with the white base of the cage; however, swarming behaviour will have to be confirmed in future studies. Field and semi-field observations have confirmed that swarming is associated with ground markers in other *An. gambiae* *sensu lato* species [13, 21, 22]. The association of *Anopheles* mosquito swarms with varied ground markers, such as open ground [7–9, 23], burnt ground and garbage heaps [7–9, 23], has been confirmed under natural field conditions. In addition, the presence of environmental features such as vegetation, termite mounds, and man-made structures such as houses has been associated with *An. funestus* swarming [7–9, 23]. These features and other landforms, such as hills, affect the natural horizon at sunset.

The FANG strain consistently exhibited higher mating rates compared to the FUMOZ strain. The highest mating success rate for the FANG strain, in this study, was approximately 80%, which is slightly higher compared to the previous insemination rates of 74.8% reported for the same strain maintained in a cylindrical cage [4]. The highest observed mating success rates for the FUMOZ strain in the control cages presented in this study were lower than those previously reported (35% vs. > 70%, respectively) [3, 6]. However, these differences may be due to differences in experimental conditions although it should also be noted that during this study period South Africa experienced major electrical supply shortages, which impacted the climatic control systems in the BDMI. These results highlight the importance of future studies to understand the impact of experimental and environmental conditions on the mating success in the FUMOZ strain.

This study has several limitations that constrain the robustness and generalisability of its conclusions. Swarming, individual mating pairs and other life table analyses (survival, etc.) were not recorded in this study. Although these variables could provide critical information on *An. funestus* mating dynamics, the objective of the work was to gather baseline information by evaluating the impact of light contrast created by a cage covering and swarm marker on the mating success in *An. funestus* females to guide future research. It is possible that multiple factors, including reducing stress, altering resting behaviour or differences in survival, can affect mating success, and these should be explored in future studies.

Another limitation was the use of a single cage per treatment per biological replicate, which raises concerns about pseudo-replication and increases the risk of type I errors during analysis. However, the difficulty in rearing and synchronising sufficient numbers of both *An. funestus* strains prevented the use of multiple cages per treatment. Reducing the number of adults per cage was avoided, as this has been shown to decrease mating success. This limitation was mitigated by increasing the number of biological repeats. Six biological replicates were conducted, and cage positions were randomised to minimise positional effects. Adult mosquito density/cage was consistent in all treatment and control cages. In addition, standardised commercially procured rearing cages were used to reduce variability associated with cage size. Additionally, the netted cage design was assumed to maintain similar airflow, microclimates, and light exposure across treatments. Limited insectary space further restricted the use of multiple cages, as placing them in close proximity would have impeded airflow and introduced additional environmental variation.

In addition, direct comparison of mating success between the FUMOZ and FANG strains (under the same cage conditions) has minor flaws. The two strains originate from different geographic locations, have different insecticide-susceptible phenotypes and were not colonised at the same time. It is therefore possible that these effects of colonisation founding might explain the observed results. An interesting research study would be to investigate if these colony-founding effects can play a role in adaptation to mating under insectary conditions.

## Conclusions

This study highlights the critical role of visual cues in influencing the mating success of *An. funestus* in laboratory settings. Manipulating the environment within rearing cages, through black horizons and black visual markers, significantly improved mating outcomes, underscoring the importance of cage design in facilitating successful copulation. Notably, the two strains tested, FUMOZ and FANG, responded differently to the same visual modifications. These differences may reflect underlying genetic or physiological variation linked to their distinct geographical origins and require further investigation. As such, standardised rearing conditions may not be universally effective across all *An. funestus* populations. To improve colony establishment and maintenance, especially when working with wild-derived strains or planning mass-rearing for release programmes, it is essential to tailor adult holding conditions to the specific characteristics of each strain. Customising the visual and environmental features of insectary cages can help accommodate subtle inter-strain differences that impact reproductive fitness. These findings have practical implications for laboratory-based vector research and colony maintenance initiatives, particularly in the context of producing large numbers of mosquitoes for experimental or operational use. They also underscore the broader need to investigate intra-species variation when designing protocols for colonising malaria vectors in captivity.

## Supplementary Information

**Additional file 1: Table S1.** Post-hoc pairwise comparisons of insemination odd ratios comparing cage cover.** Table S2.** Post-hoc pairwise comparisons of insemination odd ratios comparing visual marker.

## Data Availability

Data supporting the main conclusions of this study are included in the manuscript.

## Abbreviations

BDMI: Botha de Meillon insectary
FANG: *Anopheles funestus* strain from Angola
FUMOZ: *Anopheles funestus* strain from Mozambique
NICD: National Institute for Communicable Diseases
