# Supplementary material for: Artificial visual environments to enhance mating success in colonised Anopheles funestus
Source: Parasit Vectors. 2026 Jun 8;19:332. doi: 10.1186/s13071-026-07492-0 (PMC13470940; doi:10.1186/s13071-026-07492-0)
Supplement: Supplementary file 1 [file 13071_2026_7492_MOESM1_ESM.docx]

**Supplementary Table 1.** Post-hoc pairwise comparisons of insemination odds ratios from the generalized linear mixed model (GLMM), for *Anopheles funestus* (FANG and FUMOZ), comparing black horizon (cage specifications) within strains and strains within each cage specification.

| contrast | Strain | Odds ratio | SE | df | Lower CL | Upper CL | null | z ratio | p value | Cage Specifications |
| --- | --- | --- | --- | --- | --- | --- | --- | --- | --- | --- |
| Not Covered / Full Covered | FANG | 1.1591886 | 0.20586175 | Inf | 0.7141169 | 1.8816502 | 1 | 0.8317994 | 0.920824380457651470166524632077 |  |
| Not Covered / Bottom Covered | FANG | 0.8982323 | 0.16550715 | Inf | 0.5433820 | 1.4848143 | 1 | -0.5824773 | 0.977695380855929774277512933622 |  |
| Not Covered / Side Covered | FANG | 0.4865500 | 0.09436563 | Inf | 0.2866586 | 0.8258287 | 1 | -3.7144691 | 0.001902948369570611752976674325 |  |
| Not Covered / Top Covered | FANG | 0.6880572 | 0.12781020 | Inf | 0.4145418 | 1.1420387 | 1 | -2.0127742 | 0.259713057440007499820922021172 |  |
| Full Covered / Bottom Covered | FANG | 0.7748802 | 0.14186479 | Inf | 0.4702707 | 1.2767951 | 1 | -1.3930923 | 0.632123013456030391488127406774 |  |
| Full Covered / Side Covered | FANG | 0.4197332 | 0.08095448 | Inf | 0.2480202 | 0.7103293 | 1 | -4.5011162 | 0.000066249336879931064459015033 |  |
| Full Covered / Top Covered | FANG | 0.5935680 | 0.10959532 | Inf | 0.3587050 | 0.9822080 | 1 | -2.8250034 | 0.038067521937009130006401846913 |  |
| Bottom Covered / Side Covered | FANG | 0.5416750 | 0.10779770 | Inf | 0.3147621 | 0.9321701 | 1 | -3.0807247 | 0.017624668513895436205984879052 |  |
| Bottom Covered / Top Covered | FANG | 0.7660126 | 0.14634195 | Inf | 0.4548984 | 1.2899038 | 1 | -1.3952649 | 0.630719823283827807358647987712 |  |
| Side Covered / Top Covered | FANG | 1.4141553 | 0.28342130 | Inf | 0.8185990 | 2.4429976 | 1 | 1.7290538 | 0.415961917909799083759025961626 |  |
| Not Covered / Full Covered | FUMOZ | 1.6027001 | 0.32349476 | Inf | 0.9241379 | 2.7795070 | 1 | 2.3369074 | 0.133275066063937241978010206367 |  |
| Not Covered / Bottom Covered | FUMOZ | 0.9306340 | 0.17560279 | Inf | 0.5562167 | 1.5570902 | 1 | -0.3809880 | 0.995537596896999832374319794326 |  |
| Not Covered / Side Covered | FUMOZ | 1.1880974 | 0.22900261 | Inf | 0.7022835 | 2.0099793 | 1 | 0.8941922 | 0.899165661038250130232540868747 |  |
| Not Covered / Top Covered | FUMOZ | 0.5884892 | 0.11031971 | Inf | 0.3529064 | 0.9813354 | 1 | -2.8282798 | 0.037711983818999206263811174722 |  |
| Full Covered / Bottom Covered | FUMOZ | 0.5806663 | 0.11802388 | Inf | 0.3335324 | 1.0109165 | 1 | -2.6743573 | 0.057801559546033276504317655053 |  |
| Full Covered / Side Covered | FUMOZ | 0.7413098 | 0.15347024 | Inf | 0.4214491 | 1.3039303 | 1 | -1.4458906 | 0.597831521244460484254545917793 |  |
| Full Covered / Top Covered | FUMOZ | 0.3671861 | 0.07421508 | Inf | 0.2115657 | 0.6372757 | 1 | -4.9569277 | 0.000007096397342309934685999906 |  |
| Bottom Covered / Side Covered | FUMOZ | 1.2766537 | 0.24794388 | Inf | 0.7516163 | 2.1684531 | 1 | 1.2575949 | 0.717265390088105858623634958349 |  |
| Bottom Covered / Top Covered | FUMOZ | 0.6323530 | 0.11943409 | Inf | 0.3777552 | 1.0585437 | 1 | -2.4265443 | 0.108230515935028970275766369014 |  |
| Side Covered / Top Covered | FUMOZ | 0.4953207 | 0.09554854 | Inf | 0.2926599 | 0.8383197 | 1 | -3.6419969 | 0.002509694240789062114060925524 |  |
| FANG / FUMOZ |  | 3.2763554 | 0.59968796 | Inf | 2.2887127 | 4.6901934 | 1 | 6.4836298 | 0.000000000089541747416874965066 | Not Covered |
| FANG / FUMOZ |  | 4.5299059 | 0.89253503 | Inf | 3.0787576 | 6.6650416 | 1 | 7.6673003 | 0.000000000000017565425955741323 | Full Covered |
| FANG / FUMOZ |  | 3.3945424 | 0.64491083 | Inf | 2.3391929 | 4.9260230 | 1 | 6.4329893 | 0.000000000125118497254015231715 | Bottom Covered |
| FANG / FUMOZ |  | 8.0004717 | 1.62569672 | Inf | 5.3721898 | 11.9146102 | 1 | 10.2337568 | 0.000000000000000000000001399815 | Side Covered |
| FANG / FUMOZ |  | 2.8022376 | 0.53294394 | Inf | 1.9302745 | 4.0680927 | 1 | 5.4179747 | 0.000000060277948331744055808154 | Top Covered |

**Supplementary Table 2.** Post-hoc pairwise comparisons of insemination odds ratios from the generalized linear mixed model (GLMM), for *Anopheles funestus* (FANG and FUMOZ), comparing visual marker (cage specifications) within strains and strains within each cage specification.

| contrast | Strain | odds_ratio | SE | df | Lower CL | Upper CL | null | z ratio | p value | family | Cage_Specifications |
| --- | --- | --- | --- | --- | --- | --- | --- | --- | --- | --- | --- |
| No Marker / Top Marker | FANG | 1.2407717 | 0.26189728 | Inf | 0.7214244 | 2.1339928 | 1 | 1.022065145 | 0.73650765299917109185656727277 | Cage within Strain (Tukey) |  |
| No Marker / Bottom Marker | FANG | 1.0682895 | 0.22971002 | Inf | 0.6148666 | 1.8560812 | 1 | 0.307212715 | 0.98994665771149992217203816836 | Cage within Strain (Tukey) |  |
| No Marker / Side Marker | FANG | 1.3016685 | 0.27642350 | Inf | 0.7543378 | 2.2461302 | 1 | 1.241503981 | 0.60035142599711688315977653474 | Cage within Strain (Tukey) |  |
| Top Marker / Bottom Marker | FANG | 0.8609879 | 0.18077638 | Inf | 0.5020384 | 1.4765808 | 1 | -0.712859756 | 0.89204293924211541799706992606 | Cage within Strain (Tukey) |  |
| Top Marker / Side Marker | FANG | 1.0490798 | 0.21730851 | Inf | 0.6161646 | 1.7861596 | 1 | 0.231306820 | 0.99564311813532291317585531942 | Cage within Strain (Tukey) |  |
| Bottom Marker / Side Marker | FANG | 1.2184605 | 0.25741624 | Inf | 0.7081110 | 2.0966290 | 1 | 0.935268871 | 0.78587176215696996628423676157 | Cage within Strain (Tukey) |  |
| No Marker / Top Marker | FUMOZ | 0.9045552 | 0.17295520 | Inf | 0.5534838 | 1.4783092 | 1 | -0.524631063 | 0.95316841295954057855510654917 | Cage within Strain (Tukey) |  |
| No Marker / Bottom Marker | FUMOZ | 0.4891974 | 0.09105375 | Inf | 0.3032614 | 0.7891347 | 1 | -3.841366735 | 0.00070641895006329225026320273 | Cage within Strain (Tukey) |  |
| No Marker / Side Marker | FUMOZ | 0.9040322 | 0.17081672 | Inf | 0.5563774 | 1.4689205 | 1 | -0.533952842 | 0.95080570170118194894826046948 | Cage within Strain (Tukey) |  |
| Top Marker / Bottom Marker | FUMOZ | 0.5408154 | 0.09872697 | Inf | 0.3383551 | 0.8644211 | 1 | -3.367134050 | 0.00422800598924832904401682754 | Cage within Strain (Tukey) |  |
| Top Marker / Side Marker | FUMOZ | 0.9994218 | 0.18539080 | Inf | 0.6205626 | 1.6095780 | 1 | -0.003117917 | 0.99999998911327048212172030617 | Cage within Strain (Tukey) |  |
| Bottom Marker / Side Marker | FUMOZ | 1.8479908 | 0.33289476 | Inf | 1.1633652 | 2.9355099 | 1 | 3.409033152 | 0.00364402058449597721079271651 | Cage within Strain (Tukey) |  |
| FANG / FUMOZ |  | 8.0251553 | 1.65490564 | Inf | 5.3570180 | 12.0221955 | 1 | 10.099087125 | 0.00000000000000000000000557588 | Strain within Cage (Tukey) | No Marker |
| FANG / FUMOZ |  | 5.8505493 | 1.15522751 | Inf | 3.9730276 | 8.6153259 | 1 | 8.946465851 | 0.00000000000000000036704544419 | Strain within Cage (Tukey) | Top Marker |
| FANG / FUMOZ |  | 3.6749261 | 0.72202454 | Inf | 2.5004047 | 5.4011584 | 1 | 6.624480702 | 0.00000000003484710365927473809 | Strain within Cage (Tukey) | Bottom Marker |
| FANG / FUMOZ |  | 5.5736149 | 1.09593671 | Inf | 3.7911039 | 8.1942315 | 1 | 8.737470494 | 0.00000000000000000238387928141 | Strain within Cage (Tukey) | Side Marker |
